# Scientific Assessment of the Welfare of Trapped Mammals—Key Considerations for the Use of the Sharp and Saunders Humaneness Assessment Model

**DOI:** 10.3390/ani12030402

**Published:** 2022-02-08

**Authors:** Ngaio J. Beausoleil, Sandra E. Baker, Trudy Sharp

**Affiliations:** 1 Animal Welfare Science and Bioethics Centre, School of Veterinary Science, Massey University, Palmerston North 4410, New Zealand; 2 Wildlife Conservation Research Unit, Department of Zoology, University of Oxford, Tubney House, Oxfordshire OX13 5QL, UK; sandra.baker@zoo.ox.ac.uk; 3 Vertebrate Pest Research Unit, NSW Department of Primary Industries Tocal Agricultural Centre, Paterson, NSW 2421, Australia; trudy.sharp@dpi.nsw.gov.au

**Keywords:** animal welfare assessment, humaneness, pest control, invasive animals, trapping, mammals, five domains

## Abstract

**Simple Summary:**

The use of traps is key to the success of many wildlife management programs but the species trapped, type of trap used and its application will influence the impacts it has on animal welfare. Scientific assessment of the impacts of trapping on mammal welfare is necessary to justify the use of traps, aid trap selection, improve trap performance and develop international standards. The Sharp and Saunders humaneness assessment model was developed for the purpose of assessing the relative humaneness of a range of pest animal control methods and has been used to assess the welfare impacts of trapping on various mammal species. The model is based on the established Five Domains model, the structure of which represents the understanding that an animal’s welfare state arises due to the sum of its mental experiences which may include pain, breathlessness, thirst or fear, among many others. Here we make key recommendations for those wishing to apply the Sharp and Saunders model to scientifically assess the welfare impacts of traps. Consideration of these points will help optimize the value of information produced using the model to support ethical wildlife management practice and policy and retain social acceptance of management programs that involve trapping.

**Abstract:**

Scientific assessment of the impacts of trapping on mammal welfare is necessary to inform cost-benefit analyses of using traps in wildlife management, improve trap performance and trapping processes and develop international trap standards. The Sharp and Saunders humaneness assessment model was developed specifically for assessing welfare impacts in vertebrate wildlife management and has been used to assess the impacts of trapping various mammals. It is a specific version of the more general Five Domains model for welfare assessment which is based on the understanding that welfare state reflects the sum of the animal’s mental experiences. Our experience of applying the Sharp and Saunders model allows us to make key recommendations for those wishing to use it. First, the exact parameters of the trapping scenario to be assessed must be decided. Second, assessments should be based on published data, as well as integrating both scientific and practitioner expertise to provide rigorous and relevant outcomes. Third, conclusions about welfare impacts should be based on the appropriate indicators. As far as is possible, mental experiences should be inferred using animal-based indicators, and some representation should be provided of the scorers’ confidence in the data on which assessment is based. Careful consideration of these points will help optimize the value of information produced using the model for wildlife management decision-making.

## 1. Scientific Assessment of Animal Welfare Is Important to the Success of Wildlife Management Programs

The use of traps is integral to the success of many wildlife management programs around the world. Various types of traps exist, and they can be categorized as restraining or killing traps. Restraining traps, which are designed to hold but not kill the animal, include cage or box traps, snares, net traps, glue boards, leg-hold or foot-hold traps. Killing traps include body-grip traps, snap (or break-back) traps and electrocution traps. The choice of trap relates to the objective of the program and the trapping activity, the environment, the mammal species targeted and features of the population and individual animals [1,2,3]. The type of trap employed, and its specific application, will influence its impacts on the welfare of trapped individuals and other affected animals [4,5]. 

Robust scientific assessment of the impacts of trapping on mammal welfare is necessary for various reasons. Such information is needed to inform cost-benefit analyses of using traps in wildlife management, as well as to improve trap performance and trapping processes, and develop international trap standards [6,7]. Any welfare impacts must be considered alongside the effectiveness, cost, ease of use, human safety, non-target animal impacts and social acceptability of the trapping method [8]. In some programs, traps are used to kill unwanted wild animals, directly or indirectly (by restraining the animal for killing using another method). In such cases, the ultimate goal of welfare assessment is to identify and minimize negative impacts on the animal before irreversible loss of consciousness and death [9,10]. In other cases, certain restraining traps are used to facilitate management of valued animals through activities such as health monitoring and vaccination, translocation, collection of demographic and other research data and non-lethal population management using fertility control, (e.g., [11,12]). In these contexts, identifying and minimizing welfare impacts is additionally important because such impacts can also hinder achievement of the primary goals of the work, which may be conservation, disease management, research or monitoring [10].

More broadly, explicit and genuine consideration of the welfare of managed wild animals is critical to maintaining societal acceptance of such activities, as sectors of the public become increasingly aware of, and concerned about, animal welfare and wildlife conservation [13,14,15,16]. Thus, transparent and rigorous, science-based systems for assessing the welfare impacts of mammal trapping are critical to the future success of wildlife management programs.

## 2. The Sharp and Saunders Humaneness Assessment Model Is Based on the Five Domains Model and Facilitates Systematic, Holistic, Data-Based Assessments of Relative Welfare Impacts of Trapping

The Sharp and Saunders humaneness assessment model [17,18] was developed specifically for assessing welfare impacts in vertebrate wildlife management and has been used to evaluate the impacts of trapping of various mammal species (Table 1). The Sharp and Saunders model is a specific version of the more general ‘Five Domains model for welfare assessment’, which evolved from its original use in research, teaching and testing and is now widely applied to the systematic assessment of animal welfare states in a range of contexts, (e.g., [12,19,20,21,22,23]). 

### 2.1. Animal Welfare and the Five Domains Model for Welfare Assessment

The Five Domains model is based on the understanding that an animal’s welfare state reflects the sum of its various mental (affective) experiences at a particular point in time. In other words, animal welfare is now commonly considered to describe how the animal itself is experiencing its world and life [27,28]. While not all animals are considered capable of affective experiences (i.e., sentient), in some legal jurisdictions, mammals are explicitly deemed to be sentient and their welfare is protected to some degree e.g., European Union via the Treaty of Lisbon (2008), French Civil Code (2015), New Zealand Animal Welfare Act (2015), Australian Capital Territory Animal Welfare Act (2015) and OIE Global Animal Welfare Strategy (2017). 

Mental experiences arise due to processing of sensory information by the animal’s nervous system. Sensory receptors gather information about the outside environment (e.g., visual or olfactory signals about a predator or conspecific) and about the animal’s internal physical state (e.g., body water levels, tissue damage and respiratory function) [29]. Processing of this information by the nervous system, in a way that is specific to the species and individual, leads to generation of mental experiences, some of which matter to the animal [30]. Such ‘affective’ experiences are negative or positive, and this valence influences behavioral and physiological responses in predictable ways [31,32]. Importantly when considering the impacts of trapping, negative or unpleasant mental experiences such as thirst, hunger, pain, breathlessness and fear act as signals to the animal to respond, in a specific way, to try to alleviate or rectify the underlying problem [29]. Such negative experiences are detrimental to an animal’s current state of welfare (although they may have survival benefits in some situations). Unpleasant experiences that cannot be effectively rectified through behavioral and physiological responses, and so persist (e.g., thirst that cannot be slaked by drinking or persistent fear associated with inescapable restraint or capture), will have a greater detrimental impact on welfare state than short-lived experiences or those over which the animal has some control [27].

However, mental experiences cannot be measured directly, so must be cautiously inferred from observable indicators of the animal’s physical or physiological state or its behavior, which is permissible because of our knowledge of the links described above. This understanding of animal welfare, and the relationship between physical state and mental experiences, is reflected in the structure of the Five Domains and thus Sharp and Saunders models. These models facilitate systematic organization of the observable/measurable evidence and require interpretation of that evidence in terms of the animal’s likely mental experiences [28]. 

**Figure 1:**
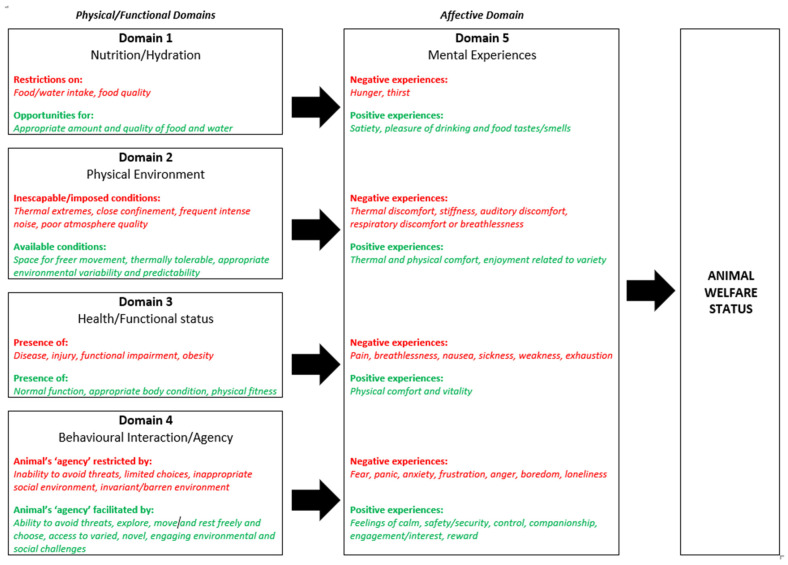
General structure of the 5 Domains model including some examples of how physical/functional impacts on the animal may relate to specific negative (and positive) mental experiences. Observable evidence of physical/functional states (welfare indicators) should be collated in Domains 1 to 4 and the associated mental experiences inferred in Domain 5. In Domain 4, ‘agency’ refers to the animal’s engagement in voluntary, goal-directed behavior. Note that the same/similar mental experiences can arise due to impacts in different physical/functional domains (e.g., breathlessness) could arise due to impaired respiration during suffocation (Domain 3) and due to poor atmosphere quality (Domain 2); exhaustion could arise due to starvation (Domain 1) or due to prolonged struggling when inescapably restrained (Domain 4). Adapted from Mellor and Beausoleil, 2015 [28].

Briefly, observable evidence of physical/functional states (welfare indicators) is collated in four Domains, which represent the animal’s nutritional and hydration status (Domain 1), its physical and sensory environment (Domain 2), its health and functional state (Domain 3) and its behavioral interactions with other animals including humans (Domain 4). In Domain 4, ‘agency’ refers to the animal’s engagement in voluntary, goal-directed behaviors, and negative impacts can arise when agency is restricted, for example when the animal is unable to escape confinement, restraint or close contact with humans or predators. The fifth Domain reflects the mental experiences likely to arise due to impacts in those four physical/functional Domains (Figure 1). Potential welfare indicators include measures of physical health and condition and behavioral, physiological and neurophysiological responses. They should be used to infer not only the occurrence but also the type (e.g., pain versus breathlessness versus thirst), intensity and duration of specific affective experiences that contribute to the animal’s overall welfare state.

### 2.2. Application of the Sharp and Saunders model to Assess Trap Impacts

The Sharp and Saunders model focuses the users’ attention on the negative impacts of wildlife management procedures such as trapping and poisoning [26]. The model can be used to assess the relative humaneness of both lethal and non-lethal traps by considering the impacts of any processes leading up to capture (non-lethal components; Part A) separately from the action that directly brings about death (Part B). Thus, Part A is used to assess all impacts of non-lethal trapping or the non-killing aspects of lethal trapping. In particular, non-killing aspects of lethal trapping include any impacts of the trap’s presence in the animal’s environment prior to capture [24]. Evaluation is usually undertaken by a panel comprising expertise of various kinds (see Section 3.2 below). 

**Figure 2:**
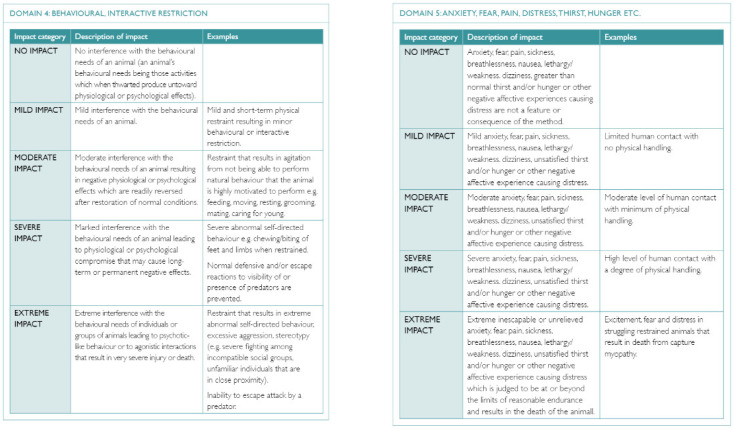
Examples of the impact scales (Domain 4 and Domain 5) to be used as a reference by panel members when applying the Sharp and Saunders model [18]. Reprinted from A model for assessing the relative humaneness of pest animal control methods. Second edition. (pp. 47–48), by T. Sharp and G. Saunders, 2011, Australian Government Department of Agriculture, Fisheries and Forestry. Reprinted with authors’ permission.

**Figure A1:**
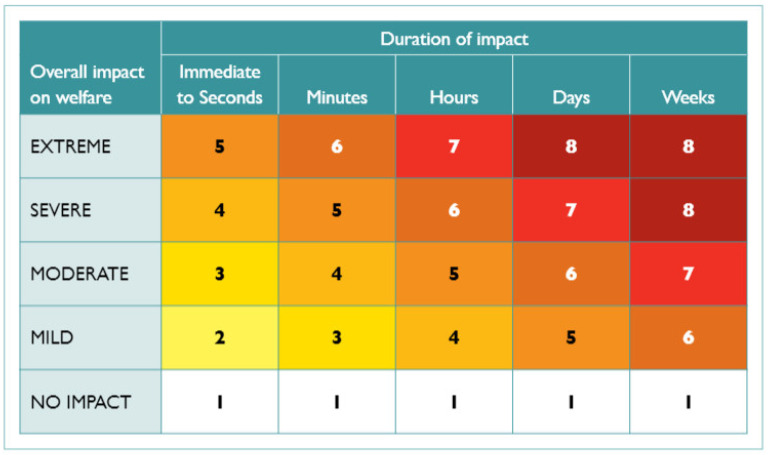
Overall welfare impacts of non-kill traps or of the non-lethal components of kill traps (Part A). Reprinted from A model for assessing the relative humaneness of pest animal control methods. Second edition. (p. 49), by T. Sharp and G. Saunders, 2011, Australian Government Department of Agriculture, Fisheries and Forestry. Reprinted with authors’ permission.

In Part A, impacts are considered in each of the five Domains and the degree/intensity of impact (score) is assigned with reference to a set of impact scales (one for each Domain) to facilitate more consistent scores among panelists (e.g., Figure 2; [18]). The impact in Domain 5 arises from impacts in Domains 1 to 4 and is usually the highest of those scores. Ultimately, an overall impact score is assigned which usually represents the score assigned in Domain 5, as mental experiences are deemed to be most relevant to welfare state—a higher overall impact score (Domain 5) represents a greater number and/or intensity of unpleasant experiences for the animal. The total duration of the non-lethal impacts is then estimated (immediate to seconds, minutes, hours, days and weeks), and the intensity and duration are integrated using a scoring matrix to assign an overall grade ranging from 1 to 8 (Appendix A Figure A1; [18]). The higher the overall grade, the more intense and/or long-lasting are any welfare impacts associated with the non-lethal components of trapping. The welfare impact of a non-lethal/restraining trapping method can be represented entirely by its Part A score; Part B of the model need not be applied.

**Figure A2:**
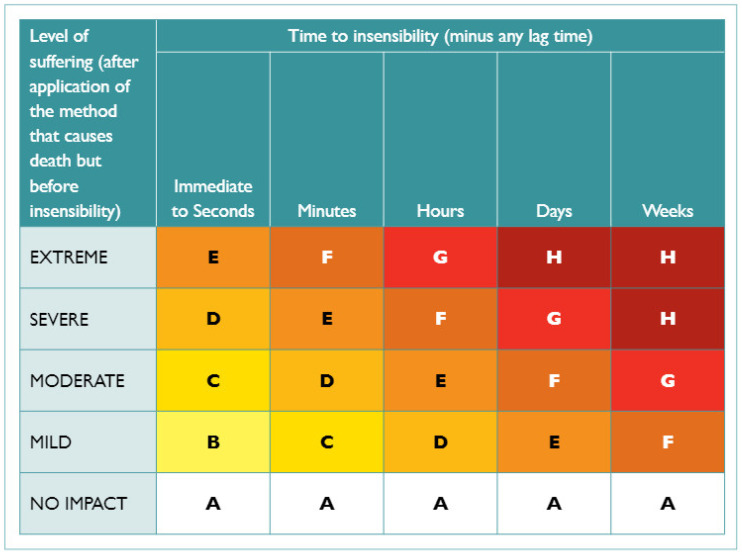
Overall impacts of the mode of death for kill traps (Part B). Reprinted from A model for assessing the relative humaneness of pest animal control methods. Second edition. (p. 52), by T. Sharp and G. Saunders, 2011, Australian Government Department of Agriculture, Fisheries and Forestry. Reprinted with authors’ permission.

For lethal trapping, Part B of the model is applied in addition to Part A, to evaluate the intensity and duration of welfare impacts associated with the killing method itself, including any handling required. Here, the intensity of suffering (integration of all unpleasant experiences occurring: No impact, Mild, Moderate, Severe and Extreme suffering) is assigned using an impact scale different from that used in Part A [see 18]. The duration of any such suffering is estimated as the time from which the method begins to have an impact (such as when a wolf becomes caught in a killing snare or a rat is struck by a snap trap) to the point at which consciousness is irreversibly lost; after this point, no affective experiences are possible. These components are combined using a separate scoring matrix to assign an overall score ranging from A to H (Appendix A Figure A2)—the higher the overall score, the greater (i.e., more intense and/or longer-lasting) any suffering associated with the killing method is deemed to be. Thus, any lethal trapping method can be ranked according to its Part A (non-lethal components; 1–8) and Part B (lethal components; A–H) scores. 

Likewise, if a lethal method (e.g., concussive blow to the head, cervical dislocation, injection of a lethal agent) is applied to the live-trapped animal, Part A and Part B assessments can be applied separately for the two components. If there are various options available for killing live-trapped animals, multiple assessments may be conducted to determine which killing method is the most humane. Box 1 provides an example of the relative welfare impacts of various methods of lethally controlling wild dogs in Australia, including evaluation of various restraining traps and subsequent options for killing. Alternatively, if the live-trapped animal is instead subjected to subsequent non-lethal procedures such as blood sampling, medical treatment or translocation, separate assessments may be conducted, again using Part A.

Evaluations are often performed with reference to a standard operating procedure for trapping in the specific context and informed by review of the relevant scientific literature by all panelists as well as by panelists’ personal experience of the procedures under assessment. Generating panel-level outcomes has been achieved in various ways, usually through panelists undertaking independent scoring followed by development of some form of consensus through discussion (e.g., [18,24]) or by representation of the range of scores within the panel, (e.g., [33]). Aiming for group consensus through discussion of the likely welfare impacts in each Domain and overall should improve the reliability of the outcomes and encourage stakeholder acceptance of the results.

Box 1 **An example of the relative welfare impacts of various methods of lethally controlling wild dogs in Australia.**

**Figure 3:**
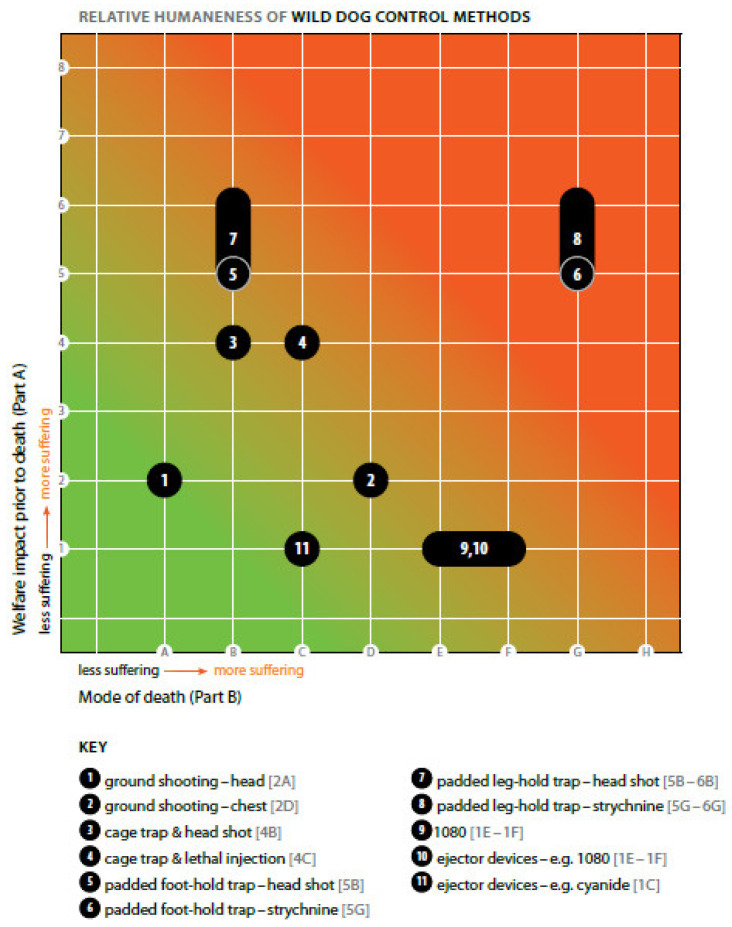
Summary of the relative welfare impacts of various methods of lethally controlling wild dogs (*Canis familiaris*) in Australia, including various restraining traps, using the Sharp and Saunders humaneness assessment model. Reprinted from A model for assessing the relative humaneness of pest animal control methods. Second edition. (p. 124), by T. Sharp and G. Saunders, 2011, Australian Government Department of Agriculture, Fisheries and Forestry. Reprinted with authors’ permission.

Summary figure (Figure 3) showing the relative welfare impacts of various methods of lethally controlling wild dogs (*Canis familiaris*) in Australia, including various restraining traps, using the Sharp and Saunders humaneness assessment model [18]. These assessments were undertaken on the protocols outlined in Standard Operating Procedures (SOP) and it was assumed that traps were checked every 24 h. Only impacts on the target animal were assessed and the scores represent consensus reached by the evaluation panel. The details of each assessment and a brief summary of the justification of the impact scores are shown in Table 2 and Table 3—see [18] for references.

These results illustrate the versatility of the model, in that the relative welfare impacts of diverse control methods can be compared. Additionally evident is the importance of clearly defining the scenario to be assessed and the value of being able to assess the non-lethal (Part A) and lethal (Part B) aspects of trapping separately. For example, the impacts of capture/restraint in a padded foot-hold or leg-hold trap are moderate to severe when applied according to the SOP (i.e., current best practice). However, the method of killing the trapped animals has a profound effect on the overall welfare impact: killing by head shot causes much less additional welfare impact (suffering) than ingestion of strychnine poison. Confidence scores were not generated for these assessments.

**Table 2:** Part A assessments of three types of restraining (non-lethal) traps used for wild dogs (*Canis familiaris*) in Australia. Summarized from data available at pestsmart.org.au (accessed 18 January 2022).

Trap	Domain 1	Domain 2	Domain 3	Domain 4	Domain 5	Duration	Impact Score
Padded foot-hold traps (e.g., Victor Soft Catch #3)	Mild (No food/water for ≤24 h)	Mild (Assumes fair weather and in shade)	Mild–Moderate (Mostly minor skin lacerations; Some leg dislocations, tooth/mouth injuries)	Moderate (Stress hormone levels high, struggling, disruption of natural behaviours)	Moderate (Anxiety, fear, frustration due to restraint, pain from injuries and struggling)	Hours	5
Padded leg-hold traps (e.g., ‘off the shelf’ padded Lanes Dingo trap)	Mild (No food/water for ≤24 h)	Mild (Assumes fair weather and in shade)	Moderate–Severe (Heavy traps tend to catch higher on leg; Leg fractures and amputations, tooth/mouth injuries)	Moderate (Stress hormone levels high, struggling, disruption of natural behaviours)	Moderate–Severe (Anxiety, fear, frustration due to restraint, pain from significant injuries and struggling)	Hours	5–6
Cage trap	Mild (Food bait but no water for ≤24 h)	Mild (Assumes fair weather and in shade)	Mild (Minor injuries; tooth, mouth, nose)	Moderate (Some stress due to restraint, struggling, disruption of natural behaviours)	Mild (Anxiety, distress due to restraint)	Hours	4

**Table 3:** Part B assessments of three methods of killing restrained wild dogs (*Canis familiaris*) in Australia. Summarized from data available at pestsmart.org.au (accessed 18 January 2022).

Method	Suffering	Duration	Impact Score
Shooting (head)	Mild (Approach of human will cause some distress)	Immediate—Seconds	B
Strychnine	Extreme (Nervousness, stiffness, progressively more frequent and intense tetanic seizures, extensor rigidity, hyperthermia, death due to exhaustion or asphyxiation)	Hours	G
Lethal injection	Mild (Approach of human will cause some distress; some pain associated with intramuscular injection)	Minutes	C

### 2.3. Advantages of the Sharp and Saunders Model for Assessing Trap Impacts on Welfare

The Sharp and Saunders model facilitates systematic, data-based, transparent and holistic assessment of the welfare impacts of trapping and other wildlife management procedures [34]. Importantly, the structure of the model encourages users to interpret the observable/measurable data in terms of what it means to the animal itself i.e., the likely affective states experienced, which is consistent with a contemporary understanding of animal welfare. The systematic nature of the assessment allows those wishing to apply the outcomes to see exactly how the scores were generated and the data upon which those scores were based. In addition, the structure of the model encourages users to look for, and organize, evidence of a wide range of physical impacts and their associated mental experiences and to consider ways to specifically mitigate those impacts when developing novel or modified trapping methods [24,35]. The model also assists in identifying any gaps in knowledge that can be addressed by future research to improve subsequent welfare assessments [24,34].

The model is versatile and can be applied to assessments of a range of trapping procedures and mammal species, and separate evaluations in Part A and B allow assessment of both lethal and non-lethal traps [18]. This versatility is demonstrated by comparisons of a diverse range of pest management methods, including fencing, scaring, habitat damage management, translocation and live release, shooting and vertebrate toxic agents (poisons), as well as various kinds of traps, in various vertebrate species [9,18,24,25,26]—see Box 1. The relative welfare impacts of a broader range of wildlife management activities could likewise be assessed, including fertility control [11], reintroduction [36], identification marking [5] and other research or management procedures [37]. However, this may require development of more generic reference scales for judging impacts in each of Domains 1 to 5. For example, it is feasible that reintroduction, medical treatment or supplemental feeding would have some beneficial effects for target or other animals, and thus conditions/states likely to lead to positive experiences may need to be integrated into impact scales (see Mellor and Beausoleil [28] for discussion of integrating negative and positive impacts into overall scores).

The Sharp and Saunders model can be used prospectively to inform consideration of the ethical permissibility of proposed traps or new uses of existing traps as well as to retrospectively evaluate actual impacts of trapping to support selection of the most humane methods, i.e., the methods with the lowest welfare impacts [38]. However, it is important to note that, as with all assessments of welfare based on inference of mental experiences, the outcomes are inherently qualitative in nature. Ordinal, rather than interval, scales are deliberately used to assign impact scores in each Domain to ensure that the qualitative nature of the assessment is explicit. The use of interval impact scores would imply a degree of precision that is neither possible nor desirable in such assessments. For example, such scores could be used to inappropriately suggest the magnitude of differences between methods (e.g., welfare impacts of trap A are twice as bad as those of trap B) [34]. In addition, it can be difficult to compare the overall impacts of different types, intensities or durations of unpleasant mental experiences that influence welfare state. For example, is a trap that causes moderate pain for days preferable to one that causes extreme breathlessness for hours [34]? These are not questions that can be resolved by scientific investigations, and there is unlikely to be a universally acceptable answer. Thus, careful application of the model, taking the following key points into consideration, will allow qualitative comparison of welfare impacts associated with different options for achieving a particular wildlife management objective.

## 3. Key Considerations for Applying the Sharp and Saunders Model and Applications of the Outcomes of Such Assessments

Our collective experience of applying the model over the last 13 years leads us to highlight a number of key considerations for those wishing to use it. Careful application of these points will help optimize the value of the information produced when using the model for wildlife management decision-making and policy development.

### 3.1. Determining the Right Trapping Scenario for Assessment

First, time should be allocated to decide on the exact parameters of the trapping scenario to be assessed; these can strongly influence the outcomes and their value for decision-making. For example, is it constructive to assess the ‘typical’ trapping event, the best- or worst-case scenario or devise an approach which integrates the likelihood of certain events occurring within the trapped population? A common approach has been to assess trapping applied according to a best practice standard operating procedure, (e.g., [24])—and see Box 1—but users should consider how well this will represent real-world applications and outcomes. To illustrate, while restraining traps are designed to be non-lethal, they sometimes cause the death of the trapped animal, for example through dehydration and/or exposure if traps are not checked regularly enough [39] or through suffocation if the animal’s muzzle is caught on a glue trap [40]. Likewise, if a killing trap is inappropriate for the species or set incorrectly, animals can be mis-caught, causing them to be restrained and potentially injured, but not killed [41,42]. Both such scenarios will result in different welfare impacts than when the trap operates optimally and when best practice procedures are followed [24,41]. Thus, it may be valuable in some cases to assess the range of possible welfare outcomes for a given method and to compare the likelihood and effects of divergence from ‘best practice’ in practical trapping situations or to examine the likely welfare gains that could be achieved by making specific changes to best practice. In all cases, the scenario to be assessed should include detailed information on the way the trap is presented in the environment and the procedures and conditions leading up to trap capture, as well as those that occur after capture in the case of live-traps.

### 3.2. Assessments Should Be Robustly Evidenced, and Panels Include Diverse Expertise

Second, assessments should be conducted by a panel that includes both academic and practitioner expertise, to provide rigorous, relevant and credible outcomes. Importantly, assessments should be led by a facilitator familiar with the model to ensure a systematic and balanced process that adheres to the underlying principles of welfare evaluation using this framework. We have found that the best results are achieved by panels including those with expertise in the management techniques and the species involved, those with expertise in veterinary physiology and pathology, and, importantly, those with expertise in the general scientific principles of animal welfare evaluation and the specific application of the Five Domains/Sharp and Saunders models. For example, the panel for a recent assessment of the welfare impacts of rat management included experts in wildlife management, rodent management, rodent biology, animal welfare science and veterinary science and medicine [24]. Failing to include those with science/biology expertise may lead to misinterpretation of clinical signs of physiological disruption or under- or over-estimation of the significance of injuries sustained. In contrast, lack of practical management expertise can mean that results are irrelevant to control as it is carried out in the real world or fail to integrate accumulated knowledge about wild animal behavior or the probability of different outcomes in trapped populations (e.g., bodily location of trap strike), reducing the credibility and applicability of the findings.

The panel should assess each method strictly as described according to the pre-defined scenario. Assessments should be informed by the relevant scientific literature, with panelists drawing on their own particular expertise to interpret the information available in terms of welfare impacts. Data published in the literature should be reviewed (and summarized if necessary) for panelists to read in advance of the assessments. Some of these data can be taken from studies specifically aiming to assess welfare impacts, but inevitably some data must be extrapolated from studies focused on the mode of action or efficacy of the method for achieving its conservation purpose, e.g., [4,33].

As the panelists work through each assessment, they should discuss the likely welfare impacts, in the context of their experience and the literature, aiming to reach consensus by doing so. A useful approach may be for panelists to make their own individual assessments following the group discussion and then to reach consensus on a score or range of scores through further discussion [18,24]. Alternatively, individual panelist’s scores may be simply represented as the median and range of the group’s scores [33].

Importantly, when making a Part A assessment, the facilitator should lead the panelists through consideration of each Domain and a detailed summary of the data (and their sources) and the thinking upon which the impact score has been based should be recorded. Likewise, a summary of data and justification of scores should be presented for Part B assessments. A way of indicating the panelists’ confidence in the scores produced using the available data is discussed below. 

### 3.3. Inferences of Animals’ Mental Experiences Should Be Based on Appropriate Indicators and an Indication of the Panel’s Confidence in Their Scores Should Be Presented

Third, conclusions about welfare impacts should be based on the appropriate indicators. As far as is possible, trapped animals’ mental experiences should be inferred using animal-based indicators. These are indicators that represent the outcome of the animal’s perception and interpretation of what happens to it and thus provide the strongest justification for inferring mental experiences and overall welfare state. Examples include measures of pathology, physiology and behavior. In contrast, resource- and management-based indicators represent risks to the animal’s welfare (inputs) but do not provide direct evidence that the resources or management are, in fact, affecting the animal’s mental state [12]. Examples include trap mechanism, space within a cage trap or animal handling. Clearly, this approach is not always possible due to a lack of validated or practically measurable animal-based indicators. Alternatively, the relationship between the event/condition and the animal’s response is so well established that resource- or management-based indicators can be used in lieu of animal-based indicators [43].

In all applications of the model, the onus is on the user to justify inferences of specific mental experiences for the taxon being assessed, as well as to demonstrate the validity of the indicators considered to reflect those experiences [43]. To illustrate, a particular challenge for assessing the duration of suffering and thus relative humaneness of kill traps has been validating indicators of loss of consciousness [25,33]. At a minimum, providing an indication of the panel’s certainty about particular inferences and thus conclusions about welfare impacts is recommended.

Our experience suggests that in addition to presenting summaries of the data on which impact scores and durations are based, it is highly beneficial to also collect and present indications of the panelists’ confidence in their scores, particularly their scores for Domain 5: mental experiences (e.g., Table 4). For example, a wider range of impact scores among panelists usually arises when there is little information available about the effects of a particular trap and this can be represented as a lower confidence score [24]. This information allows richer interpretation of the outcomes of the assessment, as well as directing research efforts to improve future understanding where data are lacking [9].

## 4. Concluding Remarks

Scientific assessment of the impacts of trapping on mammal welfare is necessary to support ethical wildlife management practice and policy and to retain social acceptance of management programs that involve trapping. The outcomes of welfare assessments using the Sharp and Saunders model can be used to develop relative rankings of the humaneness (i.e., welfare impacts) of different options for achieving management objectives and to explore ways to circumvent and/or mitigate welfare impacts and develop more welfare-friendly methods when there are no feasible alternatives [44]. Ultimately, the findings of welfare assessments should be used to inform and justify decisions about if, when and how to implement trapping activities for ‘ethical’ wildlife management [8,38]. In addition, the information gleaned from such assessments should be applied to develop international standards for trap approval [7]. Here, we have emphasized key considerations for optimal use of the Sharp and Saunders model for assessing the welfare impacts of mammal trapping; these considerations will apply equally to applications of the model more broadly in the fields of wildlife management and research. Assembling the right complement of expertise on assessment panels, including a knowledgeable facilitator and at least one expert in the application of the model itself, along with careful selection of the scenarios to be assessed and presentation of scores reflecting the panel members’ confidence in the underpinning data, will help optimize the value of the information produced using the model for wildlife management decision-making and policy development.

## Figures and Tables

**Table 1:** Application of the Sharp and Saunders humaneness assessment model to evaluate the welfare impacts of various types of traps on different species of mammal.

Species/Taxon	Trap Type	Country	Reference
Norway rat (*Rattus norvegicus*)	Cage (live)	United Kingdom	[24]
	Snap (break-back/neck)	United Kingdom	[24]
	Glue board (live)	United Kingdom	[24]
Rodents	Cage (live)	Australia	[18] *
	Snap (break-back/neck)	Australia	[18]
	Glue board (live)	Australia	[18]
European mole (*Talpa europaea*)	Spring	United Kingdom	[25]
	Box (live)	United Kingdom	[25]
Feral cat	Cage (live)	Australia	[18]
	Foot-hold	Australia	[18]
Feral goat	Pen/Yard-type	Australia	[18]
Feral horse	Pen/Yard-type	Australia	[18]
Feral pig	Pen/Yard-type	Australia	[18]
European fox (*Vulpes vulpes*)	Cage (live)	Australia	[18]
	Padded foot-hold	Australia	[18]
	Padded leg-hold	Australia	[18]
Rabbit	Padded foot-hold	Australia	[18]
Feral/Wild deer	Single pen/yard-type	Australia	[18]
	Group pen/yard-type	Australia	[18]
Wild dog (*Canis familiaris*)	Cage (live)	Australia	[18]
	Padded foot-hold	Australia	[18]
	Padded leg-hold	Australia	[18]
Brushtail possum (*Trichosurus vulpecula*)	Padded and unpadded leg-hold	New Zealand	[26]

* For results of assessments on individual mammal species/taxa see pestsmart.org.au (accessed on 13 August 2021).

**Table 4:** Confidence scores to accompany Impact and Duration Scores [9].

Confidence Score	Level of Confidence
0	No animal data available, possible negative affective experiences inferred from human reports
1	Low confidence, more specific/detailed animal data required
2	Moderate confidence, more specific/detailed animal data would clarify
3	High confidence

## Appendix A

Matrices used to score the relative animal welfare impacts of non-lethal components of kill traps or for non-kill traps (Part A; Figure A1) and the mode of death for kill traps (Part B; Figure A2) [18].
